# Significance of men’s health in long-term survivors of allogeneic stem cell transplantation

**DOI:** 10.1038/s41409-022-01638-1

**Published:** 2022-03-25

**Authors:** Laila Schneidewind, Thomas Neumann, Nadette Peters, Jennifer Kranz, Kai A. Probst, Florian H. Heidel, Oliver W. Hakenberg, William Krüger

**Affiliations:** 1 Department of Urology, University Medical Center Rostock, Rostock, Germany; 2 Department of Internal Medicine C, University Medical Center Greifswald, Greifswald, Germany; 3 Department of Urology, University Medical Center RWTH Aachen, Aachen, Germany; 4 Department of Urology and Kidney Transplantation, Martin-Luther-University, Halle (Saale), Germany; 5 Private Urological Practice, Zweibruecken, Germany

**Keywords:** Infectious diseases, Clinical trial design

## To the Editor:

Allogeneic stem cell transplantation (aSCT) is a common treatment for a variety of hematological diseases. Advances in the transplantation practices and supportive care have led to improved outcomes and an increasing number of long-term aSCT survivors. Most deaths after aSCT occur within the first 2 years as a result of relapse, acute or chronic Graft versus host disease (GvHD), infections or other acute or subacute toxicities [1, 2]. However, patients who survive beyond these 2 years after aSCT have also an increased risk of long-term complications, which may impact on their survival and quality of life [2, 3]. Reported long-term complications in this special patient population include chronic kidney disease, viral infections, e.g., BK polyomavirus (BKPyV) associated nephropathy (BKVAN) or even hypogonadism [2, 4–10]. Unfortunately, the knowledge about chronic kidney disease, urological complications as well as infections is very limited, especially in adult aSCT and in long-term survivors (more than 5 years after aSCT) [2, 4]. Furthermore, data about men’s health including hypogonadism as well as male sexual function are even more sparse in this special patient population [10, 11].

Consequently, we conducted a prospective clinical study to assess the health status and quality of life in long-term survivors of adult aSCT (>5 years following transplantation) at our institution. This is a subgroup analysis focusing on men’s health. Regarding the primary end point, this descriptive analysis focusses on urological complications and men’s health issues, e.g. hypogonadism. Secondary endpoints included associations and risk factors of these clinical conditions, quality of life as well as the impact of men’s health issues on quality of life. To the best of our knowledge, this is the first prospective study addressing these problems in male long-term survivors of aSCT.

## Subjects and methods

### Development of the study, study population and definitions

Before starting the study, we obtained the approval of the local ethics review board at the University Medicine in Greifswald (BB 146/15 from 20. October 2015). Furthermore, the study was registered at WHO Clinical Trial Registry (Universal Trial Number UTN U1111-1176-5256). Formally, this study is a prospective uni-centric non-interventional trial and this is a subgroup analysis of all included male patients. All inclusion criteria and definitions are available via UTN number.

### Statistical analysis

For each numeric variable, the numeric distribution was preliminarily assessed by the Kolmogorov–Smirnov test. Descriptive statistics were performed with mean and standard deviation for normal distribution or with median and IQR for non-parametric data. For parametric continuous variables the Student’s *t* test was used and for parametric categorical variables the chi-square test or the Fisher exact test was used. For risk factor assessment univariate Cox regression method was used. All reported p-values were based on a two-sided hypothesis, *p* < 0.05 was considered to be significant. All statistical calculations were performed using statistical package for the Social Sciences 26.0 software (SPSS Inc., Chicago, Ill., USA).

## Results

### Demographic characterization and men’s health of the study population

This study included 20 male patients with a median age of 58.1 years (IQR 52.5–61.8). The median overall survival time following aSCT was 9.0 years (IQR 7.3–15.0). Non-Hodgkin lymphoma was the most frequent underlying disease (*n* = 7; 35.0%). Ten patients (50.0%) had a pre-existing urological disease with urolithiasis being the most frequent (*n* = 5; 25.0%). At analysis, no patient had a urological disease, except from one case of primary hypogonadism (5.0%), bacterial urinary tract infection, JC polyomaviruria, or adenoviruria. The hypogonadal patient had three symptoms of hypogonadism: loss of libido, loss of secondary body hair and hot flushes. Interestingly, the FSH level was upregulated in nine (45.0%) patients. Three patients (15.0%) had BKPyV viruria, and two patients (10.0%) had pre-existing chronic kidney disease.

Furthermore, we diagnosed eight men (40.0%) with severe erectile dysfunction with the IIEF-5 (International Index of Erectile Function) questionnaire, and 17 men (85.0%) had mild lower urinary tract symptoms in the International Prostate Symptom Score.

### Clinical associations with urological conditions

BKPyV viruria was significantly linked to pre-existing chronic kidney disease (*p* = 0.001) and creatinine >100 µmol/l (*p* < 0.001).

Due to small sample size, no associations or risk factors for primary hypogonadism were identified in statistic testing or univariate Cox regression.

Interestingly, severe ED (*n* = 8; 40.0%) is significantly associated with earlier acute GvHD (aGvHD) (*p* = 0.046), but not with chronic GvHD (*p* = 0.268). Furthermore, all those cases of aGvHD showed skin involvement.

### Quality of life

The mean Global Health Status (GHS) was 75.6 (SD 13.7). An impaired GHS under 80 was not significantly associated with primary hypogonadism (*p* = 0.317), severe ED (*p* = 0.374) or severe LUTS (*p* = 0.317), respectively.

Regarding the functional scales of EORTC-QLQ-C30, the mean physical functioning was 76.1 (SD 21.5), role functioning 83.4 (SD 20.3), emotional functioning 81.5 (SD 17.8), cognitive functioning 80.7 (SD 27.2) and social functioning 92.2 (SD 11.0), respectively. In summary, primary hypogonadism is significantly associated with impaired physical functioning and role functioning. Severe ED is significantly linked to impaired cognitive functioning and severe LUTS results in a significant impairment of all functioning scales.

**Fig. 1:**
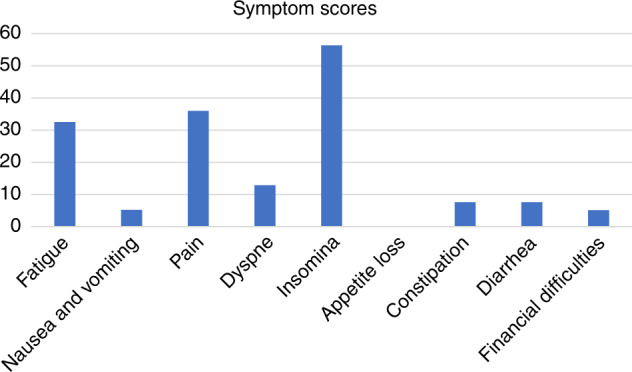
Symptom Scores in EORTC-QLQ-C30 (*n* = 30).

Regarding the symptom scales of EORTC-QLQ-C30, the mean fatigue score was 32.5 (SD 26.4), nausea and vomiting 5.2 (SD 18,6), pain 36.0 (SD 33.9), dyspnoe 12.0 (SD 25.7), insomnia 56.3 (SD 34.5), appetite loss 0 (SD 0), constipation 7.6 (SD 14.5), diarrhea 7.6 (SD 14.5) and financial difficulties 5.1 (SD 12.4), respectively. Overall, insomnia and pain are the most frequent symptoms in this patient population. Figure 1 illustrates the symptom scores in these men. Interestingly, primary hypogonadism is significantly associated with fatigue (*p* = 0.006) and dyspnoe (*p* = 0.049), while severe LUTS is significantly linked to fatigue (*p* = 0.006), pain (*p* = 0.001), dyspnoe (*p* = 0.040), insomnia (*p* < 0.001) and appetite loss (*p* < 0.001), respectively. There were no significant associations of symptoms with severe ED identified.

## Discussion

We conducted a prospective clinical study about men’s health in very long-term survivors of aSCT.

The study’s most frequent urological problem was severe ED. Interestingly, severe ED is significantly associated with aGvHD. Additionally, all episodes of aGvHD showed skin involvement. The explanation of this phenomenon is not entirely obvious, but it is described in clinical and experimental studies that fibrosis pathways are involved in the pathogenesis of ED [12]. Therefore, we suggest the hypothesis, that GvHD can also lead to a fibrosis in the cavernous body of the penis.

One major advantage of this investigation is the detailed description of the quality of life in this male population, e.g. we identified pain and insomnia as leading symptoms, which should be discussed with the patient during follow up, but we must assume that this study has several limitations, such as selection bias due to uni-centric setting and small sample size.

In summary, this study provides the first data about men’s health in long-term survivors of aSCT. We hope to sensitize physicians for this topic since it can profoundly impact the quality of life, so they will become more familiar with fertility preservation, diagnosis, and treatment of hypogonadism as well as LUTS. Unfortunately, there is also a lack of research in this area, but this is absolutely essential as well since urological problems can impair overall health, e.g. kidney function.

## Data Availability

All data are available upon request from the corresponding author.

## References

[1] Wingard WR, Majhail NS, Brazaukas R, Wang Z, Sobocinski KA, Jacobson D, et al. Long-term survival and late deaths after allogeneic hematopoietic cell transplantation. J Clin Oncol. 2011;29:2230–9. 21464398 10.1200/JCO.2010.33.7212 PMC3107742

[2] Neumann T, Peters N, Kranz J, Dräger DL, Heidel FH, Krüger W, et al. Significance of BK polyomavirus in long-term survivors after adult allogeneic stem cell transplantation. Biology. 2021;10:553. 10.3390/biology10060553. 34205390 10.3390/biology10060553 PMC8234795

[3] Torrent A, Ferra C, Batlle M, Hidalgo F, Jimenez-Lorenzo MJ, Ribera JM. Prospective follow-up of adult long-term survivors of allogeneic haematopoietic stem cell transplantation. Med Clin. 2020. 10.1016/j.medcli.2020.07.038. 10.1016/j.medcli.2020.07.038 33250187

[4] Jo T, Arai Y, Kondo T, Kitano T, Hishizawa M, Yamashita K, et al. Chronic kidney disease in long-term survi-vors after allogeneic hematopoitic stem cell transplantation: retrospective analysis at a single institution. Biol Blood Marrow Transpl. 2017;23:2159–65. 10.1016/j.bbmt.2017.08.016 28822830

[5] Pinana JL, Valcarcel D, Martino R, Barba P, Moreno E, Sureda A, et al. Study of kidney function impairment after reduced-intensity conditioning allogeneic hematopoietic stem cell transplantation. A single center experience. Biol Blood Marrow Transpl. 2009;45:21–9. 10.1016/j.bbmt.2008.10.011 19135939

[6] Liu H, Li YF, Liu BC, Ding JH, Chen BA, Xu WL, et al. A multicenter retrospective study of acute kidney injury in adult patients with nonmyeloablative hematopoietic SCT. Bone Marrow Transpl. 2010;45:153–8. 10.1038/bmt.2009.99 19430501

[7] Kagoya Y, Kataoka K, Nannya Y, Kurokawa M. Pretransplant predictors and posttransplant sequels of acute kidney injury after allogeneic stem cell transplantation. Biol Blood Marrow Transpl. 2011;17:394–400. 10.1016/j.bbmt.2010.07.010 20655388

[8] Shimoi T, Ando M, Munakata W, Kobayashi T, Kakihana K, Ohashi K, et al. The significant impact of acute kidney injury on CKD in patients who survived over 10 years after myeloablative allogeneic SCT. Bone Marrow Transpl. 2013;48:80–4. 10.1038/bmt.2012.85 22635246

[9] Hingorani S. Renal complications of hematopoietic-cell transplantation. N Engl J Med. 2016;374:2256–67. 27276563 10.1056/NEJMra1404711

[10] Haavisto A, Mathiesen S, Suominen A, Lähteenmäki F, Sorensen K, Ifversen M, et al. Male sexual function after hematopoietic stem cell transplantation in childhood: a multicenter study. Cancers. 2020;12:1786. 10.3390/cancers12071786. 32635426 10.3390/cancers12071786 PMC7408376

[11] Phelan R, Im A, Hunter RL, Inamoto Y, Lupo-Stanghellini MT, Rovo A, et al. Male-specific late effects in adult hematopoietic cell transplantation recipients: a systematic review from the Late Effects and Quality of Life Working Committee of the Center for International Blood and Marrow Transplant Research and Transplant Complications Working Party of the European Society of Blood and Marrow Transplantation. Transpl Cell Ther. 2021;29:S2666–6367. 10.1016/j.jtct.2021.10.013. 10.1038/s41409-022-01591-z PMC10316716 35523848

[12] Liu K, Cui K, Feng H, Lin H, Chen Y, Zhang Y, et al. JTE-013 supplementation improves erectile dysfunction in rats with streptozotocin-induced type I diabetes through the inhibition of the rho-kinase pathway, fibrosis, and apoptosis. Andrology. 2020;2:497–508. 10.1111/andr.12716 31610097

