# Correction: Significance of men’s health in long-term survivors of allogeneic stem cell transplantation

**DOI:** 10.1038/s41409-024-02415-y

**Published:** 2024-10-04

**Authors:** Laila Schneidewind, Thomas Neumann, Nadette Peters, Jennifer Kranz, Kai A. Probst, Florian H. Heidel, Oliver W. Hakenberg, William Krüger

**Affiliations:** 1 Department of Urology, University Medical Center Rostock, Rostock, Germany; 2 Department of Internal Medicine C, University Medical Center Greifswald, Greifswald, Germany; 3 Department of Urology, University Medical Center RWTH Aachen, Aachen, Germany; 4 Department of Urology and Kidney Transplantation, Martin-Luther-University, Halle (Saale), Germany; 5 Private Urological Practice, Zweibruecken, Germany

**Keywords:** Infectious diseases, Clinical trial design

Correction to: *Bone Marrow Transplantation* 10.1038/s41409-022-01638-1, published online 25 March 2022

In this article the author’s name Nadette Peters was incorrectly written as Nandette Peters.

The original article has been corrected.

